# Clinical Society of Maryland

**Published:** 1896-08

**Authors:** H. O. Reik

**Affiliations:** Secretary; 525 N. Howard street; Baltimore


### *SOCIETY REPORTS.*

### CLINICAL SOCIETY OF MARYLAND.

BALTIMORE, March 6, 1896.

DR. HENRY M. HURD read a paper on "Paranoia." Dr. Hurd first related a case of paranoia now under his care, and then said, in part, as follows: "In paranoia we have to deal with an original abnormal personality, developing badly and deviating more widely each year from a normal type, until finally the departure from health becomes so wide that a dangerous lunatic is developed, who requires confinement and seclusion to insure the safety of society and the welfare of the community. In the early stages of the disease the delusions relate to encroachments upon the life, health, honor, or property of the patient. Such patients are usually self-centered, and from childhood have been reserved, suspicious, and even hypochondriacal. They are generally badly developed and have the more common stigmata of degeneracy, as a want of symmetry of both sides of the face, a lack of development of the facial bones, giving rise to the protruding chin and "whopper jaw" so characteristic of the descendants of the Emperor Charles V., or unsymmetrical palpebral fissures. They are generally unduly responsive to all external disturbing influences and the development of morbid characteristics may follow comparatively slight disturbing causes. Paranoia develops as an unmistakable disease when hallucinations of the special senses give rise to actual delusions. The delusions are at first, and often for many years, those of persecution, and their character is determined by their habits of life, system of beliefs, and, above all, their antecedent mental development or education. They believe the world to be generally unfriendly to them, and seclude themselves from their fellows. Delusions of poison are very common, and many in consequence confine themselves to a very restricted diet. They do their own cooking often, and take nothing but eggs cooked in the shell, potatoes boiled in their jackets, or milk, which, the world over, is believed to be directly antagonistic to all forms of poison. Sooner or later, however, they are forced by the vividness of their hallucinations to defy their enemies, and then develop dangerous tendencies. Most of the crimes committed by the paranoiac are done at this stage of the disease. The terminal stage of paranoia is what has been happily termed by one writer the stage of transformation by which through a further elaboration of his delusions the patient finally believes he has solved the terrible secret which has heretofore clouded his whole life. He begins to believe that he is persecuted because he is a superior being, and delusions of grandeur, power, and importance replace those of persecution, so that though he may suffer still, he rejoices more than he suffers.

"Writers upon paranoia have made many different forms of this disease; for example, religious paranoia, sexual paranoia, etc., but the essential elements of the disease are the same in all instances. Whatever the form of the disease may be, they all lead to the development of dangerous impulses and threaten the peace and good order of the community where such patients dwell. The majority of crimes, especially deeds of violence, which are committed by insane men are committed by the victims of this form of disease. Personally I have little patience with medical men who, when called upon to give evidence in cases of this character, belittle the gravity of delusions of persecution. It is common to call them harmless, and to speak of these patients as peculiar, "queer," "unbalanced," but as not "insane." As a matter of fact, the delusions of the paranoiac are, next to the impulsive, blind, unreasoning fury of the epileptic, most apt to result in homicide, deeds of violence and outrageous crimes. Every case of developed paranoia should be under custody and control until such time as the stage of transformation occurs.

The time to treat the paranoiac is largely in childhood and youth. All patients who possess a neurotic precocity which is likely to terminate in paranoia should be carefully trained and educated with the hope that the inherent physical and mental defect may be removed by healthy habits of living and thinking. An effort should be made later when hallucinations are developing, and delusions are likely to follow them, to break up morbid habits of introspection by putting a stop to the devices which these patients employ to seclude themselves from the world and to cherish their morbid fancies. When self-control is lost and the patient becomes the victim of systematized delusions, little can be done except to place him under strict control. The fallacy that he is harmless at this stage has cost the lives of many excellent people, and will continue to do so unless the profession takes a more serious view of this class of cases. In the final stage little medical care is required. Such patients are usually cheerful, industrious, and when under proper control, able to contribute somewhat to their own support. Many of the useful laborers in institutions for the insane are of this class."

DISCUSSION.

DR. CHARLES G. HILL: "I am sorry that I did not arrive in time to hear all of Dr. Hurd's paper. There are a number of points in connection with paranoia that make it of interest not only to physicians but to the general public. The first of these is its diagnosis. The term may be so extended as to embrace a great deal, and as we all have some delusions, some have classed the greater part of the human race as paranoiacs. We are generally speaking of special cases, however, where these delusions are of such character as to render the patient unfit to be at large. Another point is the disposition of paranoiacs. No doubt many of them are innocent, and I have known some of the subjects of such delusions all their lives, but notwithstanding which they are able to fill their places in society. Some of these, however, suddenly become dangerous. I remember one case, a man of fifty-one or -two years of age, who, from what he considered a coolness on the part of his wife, accused her of unfaithfulness, and purchasing a pistol went gunning for his most intimate friend. It was then found necessary to confine him, and I had him under my care for five or six months, when, against my advice, he was removed from the institution. His delusions had somewhat abated, or he had at least concealed them, and he has lived in good relations with his wife ever since. On inquiry of his friends, I found that throughout his life he had shown delusions, and had been the cause of various ruptures with his friends. Without any cause he would suppose that some business friend was trying to injure him. The friend would usually try to explain, and then give him up as a "crank." Finally the occurrence mentioned above resulted, probably due to the subsidence of sexual activity on the part of his wife. Under such circumstances as these a paranoiac is always dangerous. I have a case under treatment now of a young man who thought that a prominent man of this city wanted to force him to marry his daughter. He was a man of rather low station in life, and the idea was ridiculous. He was kept at home because he talked of trying to kill the man, but he escaped from his friends, and when found was parading before the gentleman's door with a pistol in hand, and the only thing that had prevented a possible murder was that he was unacquainted with the gentleman he was gunning for. The man came out of his house while he was watching it. This goes to illustrate the dangerousness of paranoiacs. It was the only delusion this man had. The best thing to do would be to confine all paranoiacs in asylums where the régime and regular life would guard them from danger to others, if it did not benefit themselves.

DR. G. H. ROHE: There has been such a dearth of accurate description of this class of cases in English literature that I have been in the habit of recommending to my classes the reading of a novel, which describes it pretty accurately; I mean "Peter Ibbetson." There is no record which better describes a case of paranoia from the initial trouble to the delusions and ultimate result, the crime. Dr. Hurd, so far as I am aware, gives the first classical description of paranoia in English. I am glad that Dr. Hurd laid such stress on the dangers of this disease, for indeed it is like the old description of a gun, "dangerous without lock, stock, or barrel." The non-dangerous one, in my opinion, does not exist. When he passes the first stages, and arrives at that of grandeur he may be, if under observation, no longer a serious menace to others, but so long as he is under the force of delusions he is a menace to society and should be confined. I appreciate very thoroughly the disgust that Dr. Hurd feels for the physician that deliberately talks of the "harmless crank," as if because a man is not a maniac he can be allowed to go about his business, particularly if he shows some good business sense. These individuals may be the most dangerous kinds of insane persons. I am a little skeptical as to the value of treatment, even in the early stages. The difficulty is that the abnormally developed and eccentric child, and usually the precocious child, is not brought to the doctor for treatment, but is held up as a good example to others, and the doctor's advice, if it be to repress this precocity, is not usually followed; still such advice should be given when the opportunity offers.

DR. E. N. BRUSH: I agree most thoroughly with Dr. Rohe that the Society is to be congratulated on having heard this paper, but I would like to add that this is not the first of Dr. Hurd's publications on this subject. I am glad to hear Dr. Rohe speak of this novel, for I know of no better description of this disease in any text-book. To-night I passed a man on the street breathing out talk of fire and murder, and saying if I catch him I'll kill him. I turned to a shopkeeper in the neighborhood and was informed that he was a harmless "crank." I inquired further and found that for several years this man had labored under the delusion that people were putting bugs in his bed and that they crawled up his spine and disturbed his brain. Judge Harlan told me that I would be justified in arresting such a man on the street, but I did not feel like picking this man up to-night.

DR. A. K. BOND: The part of this subject that most interests me is its cause and possible prevention. I do not suppose that particular forms of insanity are inherited, but judge that it is simply some underlying brain weakness that produces one or the other of the forms of brain trouble. If that is so, then the securing of healthier infants is the way to do good, and that might be done by promoting the nutrition of the mother during pregnancy. It is known that a woman who produces an idiot at one birth may produce a perfectly healthy child at the next, if her nutrition is kept up to a high mark, so I think in these neurotic families the doctor should take more care in that direction. As to the treatment by confinement, probably the best thing we could do for the State would be the building of a sanitarium for nervous diseases where the patient who goes in is not branded for ever afterwards as insane, as is the case with a patient to either of the present State institutions. We want the benefit of hospital treatment without the usual stigma. One thing I would like to know is whether there is any particular symptom that will direct your attention to paranoia, and I would like to ask also whether in the early stages nutrition of the brain would do good towards preventing the disease, or whether it is a disease certain to advance.

DR. H. M. HURD: I think it may be said that no child is born insane, but all neurotic children are born with a predisposition to insanity, and this tendency may be eradicated by surrounding the child with good and proper influences, and by sensible systematic education. Many neurotic children escape disease because they are educated and cared for by judicious people; in the case of the paranoiac there is beginning in intrauterine life some defect, it may be in the nutrition in the brain cells themselves, but more likely in the symmetrical and harmonious development of the brain. This may be due to pressure of the bones of the skull through premature ossification, or to an unequal development of the bones of the skull or face, as a result of which the brain nutrition is interfered with and it fails to develop properly. It is to be borne in mind that while this may be so it does not necessarily follow that such a child will inevitably become paranoiac. I am sure that these inherited tendencies can be overcome by proper effort made to nourish, strengthen, and develop a congenitally weak brain. As to the appearances which suggest paranoia to the physician, I would say that he must rely upon the usual stigmata of degeneration, the lack of symmetry in the skull, or about the face, the inequality of the palpebral fissures, the character of the ear and the general shape of the lower part of the face.

DR. H. O. REIK,

Secretary.

525 *N. Howard street.*

